# Percutaneous posterior transiliac plate versus iliosacral screw fixation for posterior fixation of Tile C-type pelvic fractures: a retrospective comparative study

**DOI:** 10.1186/s12891-022-05536-x

**Published:** 2022-06-16

**Authors:** Chul-Ho Kim, Jung Jae Kim, Ji Wan Kim

**Affiliations:** 1 Department of Orthopedic Surgery, Chung-Ang University Hospital, Chung-Ang University College of Medicine, Seoul, Republic of Korea; 2 Samsong Seoul Orthopedic Clinic, Goyang, Republic of Korea; 3 Department of Orthopaedic Surgery, Asan Medical Center, University of Ulsan College of Medicine, 88 Olympic-ro 43-gil, Songpa-gu, Seoul, 05505 Republic of Korea

**Keywords:** Iliosacral screw, Trasiliac plate fixation, Pelvic bone fracture

## Abstract

**Background:**

This study aimed to compare the clinical outcomes and complications between two minimally invasive surgical techniques: percutaneous transiliac plate fixation and iliosacral (IS) screw fixation for the treatment of Tile C-type pelvic bone fractures.

**Methods:**

We retrospectively reviewed the data of 77 consecutive patients with Tile C pelvic ring injuries who underwent either percutaneous transiliac plate fixation or IS screw fixation in a single academic center between November 2007 and January 2018. We recorded patients’ demographics, surgery-related data, and postoperative surgical outcomes and compared the incidence of complications and revision surgery rates between the two groups.

**Results:**

Overall, 14 patients were included in the plate group, while 63 were included in the IS screw fixation group. No significant differences were observed in the patients’ demographics between the two groups except for a longer interval from injury to surgery (13.5 days vs. 5.4 days, *P* = 0.001). Both groups acquired fracture union in all cases. There was one case of infection requiring surgical debridement in the plating group. Notably, nerve injury (*n* = 3) and implant loosening (*n* = 5) occurred in the IS screw group, but the difference was not significant.

**Conclusions:**

Both percutaneous posterior transiliac plating and IS screw fixation in patients with Tile C-type pelvic bone fractures showed good results. We recommend IS screw fixation as the primary treatment and propose posterior plating as treatment for sacral dysmorphism and bilateral sacral alar fractures in patients with spinopelvic dissociation.

**Level of evidence:**

III

## Background

Tile C-type pelvic bone fractures refer to fractures occurring in the pelvic ring, which are both rotationally and vertically unstable [1], accompanied by complete disruption of the posterior arch of the pelvic bone. For these fractures, both anterior fixation and posterior fixation are generally required. For posterior fixation, a percutaneous transiliac plate fixation method and iliosacral (IS) screw fixation methods have gained popularity as minimally invasive techniques [2], but the choice between the two methods remains controversial.

Since the IS screw fixation technique was introduced in the late 1990s, it has become increasingly popular and has shown excellent results over the past few decades [3–5]. The advantages of IS screw fixation are as follows: it can be performed in a supine position, combined anterior fixation can be performed with ease, only a small incision is needed, it is associated with minimal blood loss, and the risk of soft tissue injuries or deep infection is low [6, 7]. However, there are also concerns associated with percutaneous IS screw fixation due to the technical demands of the procedure [3] and the possibility of iatrogenic neurovascular injuries from malpositioned screws [8]. Alternatively, posterior transiliac plate fixation allows a more intuitive implant placement and has lower neurovascular damage risk [9]; however, there are concerns regarding other postoperative complications such as infection or pressure sores [10, 11].

Several studies comparing plate fixation and IS screw fixation for the treatment of anterior and posterior pelvic ring fractures have been published. However, studies that provide a direct comparison between the two methods of posterior pelvic ring disruption are limited [2, 12]. Therefore, this study aimed to compare the clinical outcomes and complications of these two minimally invasive surgical techniques for the treatment of Tile C-type pelvic bone fractures.

## Methods

This study was approved by the Institutional Review Board of Asan Medical Center and waiver was received for the need to provide written informed consent. Data collection was performed in accordance with relevant guidelines and regulations of the committee.

### Patient selection

We performed a retrospective review of the medical records of all consecutive patients who had undergone percutaneous plate or IS screw fixation for posterior pelvic ring fractures between November 2007 and January 2018 in a university hospital. All of the pelvic bone fracture patient underwent 3D reconstruction CT scan preoperatively, and the fracture patterns were confirmed by CT images. Of these patients, we included those who 1) had undergone percutaneous transiliac plate fixation or IS screw fixation for a posterior pelvic ring fracture and 2) those who experienced a Tile C-type pelvic ring fracture. We excluded patients who 1) were diagnosed with pelvic insufficiency fractures, 2) had not undergone anterior fixation; these patients were excluded since they had relatively stable injuries, and 3) required for decompression surgeries of sacral canal, 4) with displaced sacral U-type fractures; these fractures usually required triangular fixation, which was not sufficiently stabilized with posterior plating or IS screws [13]. We did not exclude the patient who lumbosacral nerve injuries as preoperatively, or hemorrhagic shock.

### Selection of fixation method, surgical techniques, and postoperative rehabilitations

All procedures were performed by two experienced faculty surgeons specializing in pelvic trauma surgery. For patients in whom acceptable fracture reduction could be achieved for posterior pelvic ring injuries, either a percutaneous plate fixation or IS screw fixation technique was employed. For patients with sacral dysmorphism, spinopelvic dissociation with bilateral sacral alar fractures, or a high risk of nerve injury due to severe anteroposterior (AP) displacement of > 10 mm, percutaneous plating was preferred over IS screw fixation. Although we applied an individual postoperative rehabilitation protocol, weight-bearing was not permitted until postoperative 4 weeks, and patients were ambulated with non-weight-bearing or used a wheelchair. The 4 weeks after surgeries, the pelvic radiographs series including AP, inlet/outlet, and oblique view were taken and compared with previous postoperative radiographs. After confirming the consistency of fracture reduction status, the patients were allowed tolerable ambulation with gradually increasing weight-bearing.

### Percutaneous posterior transiliac plate fixation

**Fig. 1:**
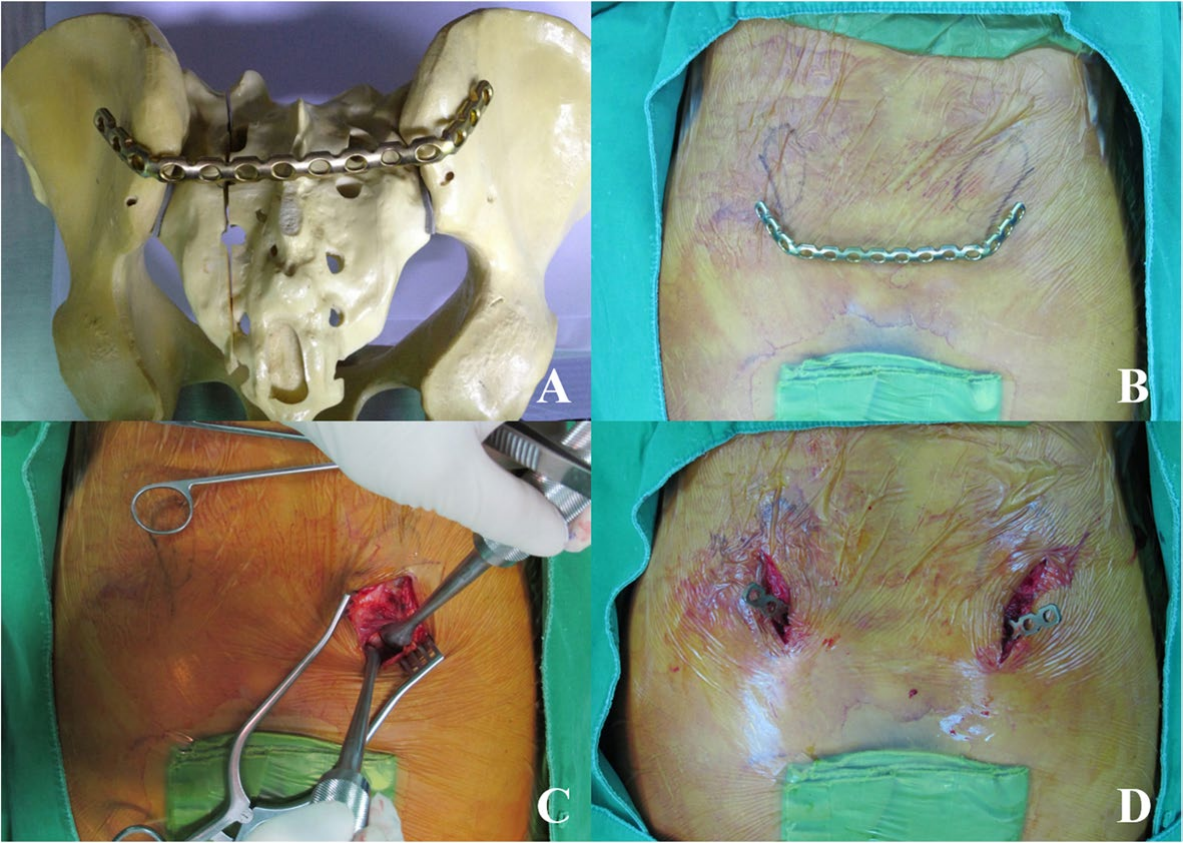
The percutaneous plate fixation technique. **A** Preparation of pre-bending in plate reconstruction is conducted using a plastic pelvis bone. **B** The incision is drawn at the lateral side of the posterior superior iliac spine. **C** The posterior superior iliac spine is exposed following the dissection of the superficial fascia. **D** The plate is inserted through the subcutaneous tunnel, and the plate is located on the dorsal side of both ilia

With the patient in the prone position, 3-cm dual vertical incisions were made lateral to the posterior superior iliac spine (PSIS). After the PSIS was exposed, the superficial fascia was incised along the direction of its fibers and retracted. The gluteal muscles were stripped away from the outer plate of the ilium, and pre-bent 3.5-mm reconstruction plates were inserted just below the posterosuperior iliac spine to one side following resection of the S3 spinous process under fluoroscopy. The plate was inserted into the opposite side through the subcutaneous tunnel and placed on the dorsal side of both ilia. We used at least 3 screws for side, minimum 6 screws were inserted entirely. Finally, the screws were positioned to penetrate the bony cortex on both sides. The details of process for surgical technique of transiliac plate fixation is shown in Fig. 1 [14–16].

### IS screw fixation

**Fig. 2:**
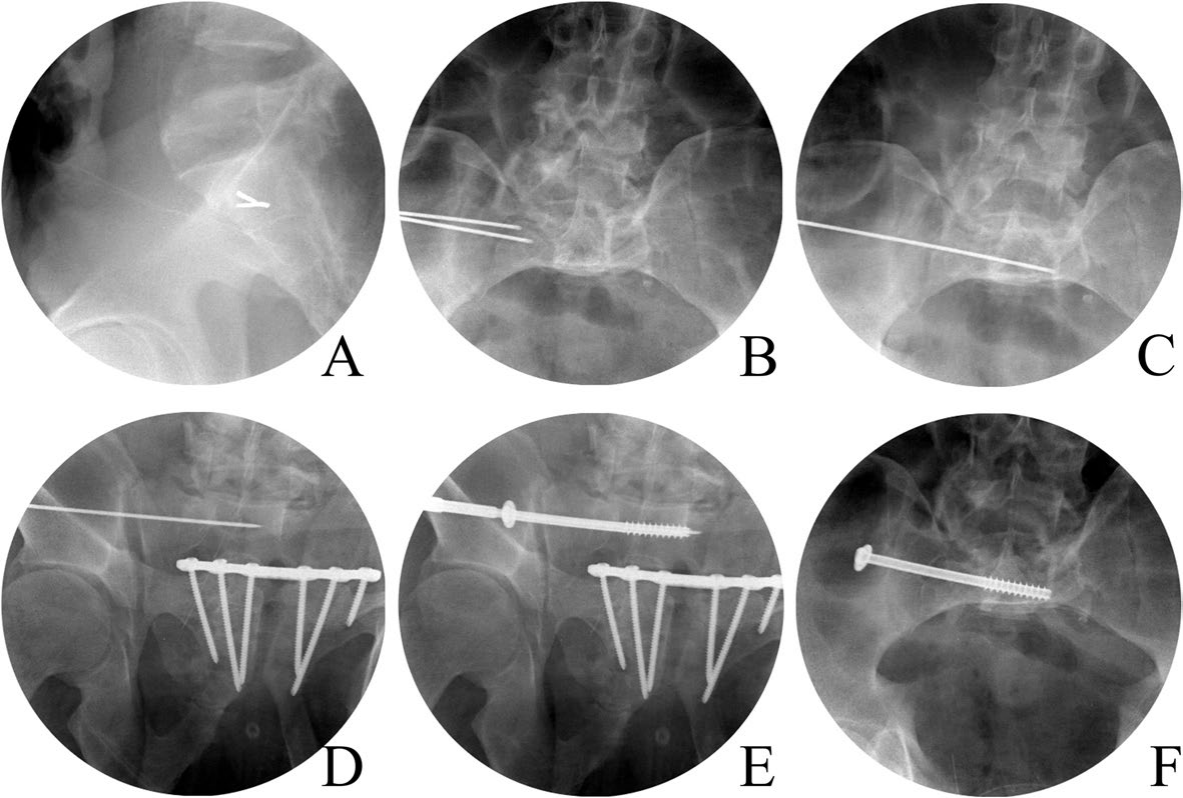
The IS screw fixation technique on C-arm images. **A** The guidewire was inserted through safe zone confirming with C-arm lateral view. **B** the guidewire was advanced through outer, inner cortex of ilium, and sacrum outer cortex. **C** The position of guidewire was confirmed in pelvis inlet view, and **D** outlet view. **E** Inserted screw was checked to avoid violation of S1 foramen on pelvis outlet view. **F** screw breach in AP direction was checked on pelvis inlet view

The patients were placed in a supine position on a transparent surgical table. A pad was placed under the lumbar region, and a small incision (1 cm) was made after identifying the safe zone on the lateral side of the pelvis. A guidewire was inserted through the safe zone and confirmed with C-arm inlet and outlet views [4, 8]. After confirming that the cannula was inserted through the incision as far as the ilium, the cannula was positioned parallel to the upper portion of the S1 vertebra along the superior margin under C-arm guidance. The guidewire was inserted from the ilium through the sacrum cross the middle of sacral body carefully so that it did not breach the sacral foramina, anterior and posterior to the cortex of the sacral body. Following Kirschner wire insertion, cannulated drill holes were made and 7.0- or 7.3-mm cannulated screws were introduced. If the fracture was considered highly unstable, the screws were inserted in the S2 vertebra in the same manner. The details of process for surgical technique of IS screw fixation is shown in Fig. 2.

### Data collection and statistical analysis

From the medical records, we extracted the demographic data of both groups, including age, sex, body mass index, injury mechanism, time to surgery following injury, and follow-up duration. The perioperative surgery-related outcomes were assessed: fixation method of the anterior pelvic ring, volume of blood loss, operation time, and length of hospital stay. We reviewed the postoperative outcomes, including union rate, incidence of delayed union, incidence of iatrogenic nerve injury of the L5 or S1 nerve after posterior fixation, rates of vessel injury, incidence of implant loosening, postoperative infection rates, and need for revision surgery. The Majeed functional score, and Harris hip score were also reviewed in both two groups.

To compare variables between the two groups, the Mann–Whitney U tests were used for continuous variables, while the chi-square test or Fisher’s exact test was used to evaluate categorical variables, after verifying the assumption that the data follows a normal distribution. As all continuous variables were not normally distributed, the Mann–Whitney U tests were used to evaluate these variables in the current study. Moreover, the chi-square test was used to compare the two fixation methods of anterior instability, while the Fisher’s exact test was used to evaluate other categorical variables after verifying the normality of its distribution. All statistical analyses were performed using PASW Statistics version 18.0 (IBM Corp., Armonk, NY, USA). A *p*-value of < 0.05 was considered significant.

## Results

**Fig. 3:**
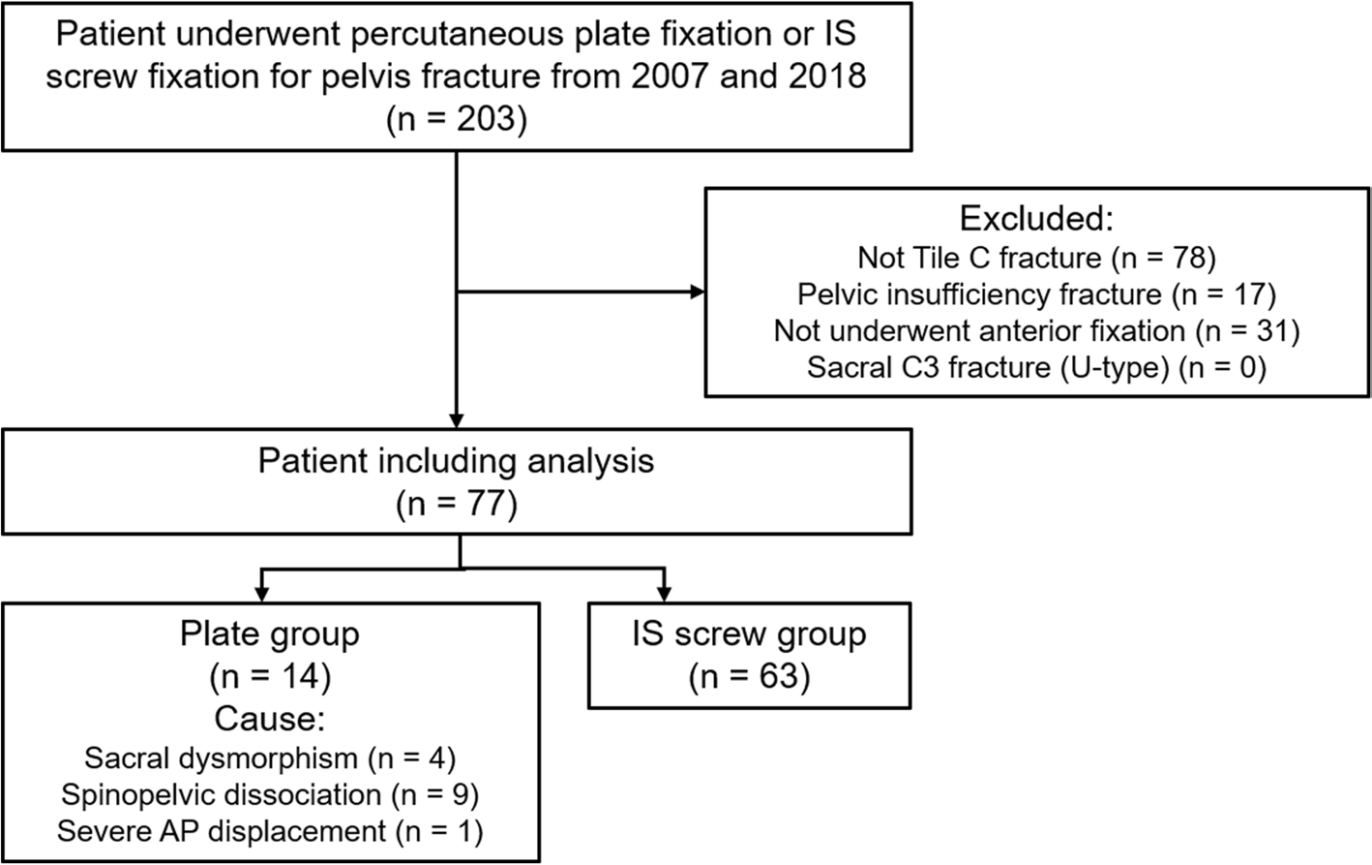
The flowchart of the patient exclusion process is based on the study criteria

Among the 203 patients initially screened, 77 were eligible for the analysis (Fig. 3). Of the total study population, 14 patients were included in the plate group and 63 in the IS screw group. Over 31 patients had Tile C1-type (unilateral type) fractures, 36 had Tile C2-type (bilateral type), and 10 had Tile C3-type (bilaterally vertically unstable type). The causes of plate fixation were as follows: dysmorphism (*n* = 4), spinopelvic dissociation with bilateral sacral alar fractures (*n* = 9), and severe AP displacement of more than 10 mm (*n* = 1). The mean age of all included patients was 46.5 years (range, 13–84 years; standard deviation [SD], 16.3). Of the 77 patients, 46 were men and 31 were women. The mean follow-up duration was 36.4 months (range, 1.5–131.9; SD, 32.1); the detailed follow-up durations were 23.9 months in the plate group and 39.4 months in the IS screw group (*p* = 0.494). A comparison of demographic data between the two groups is shown in Table 1. No significant differences were observed in any of the variables between the two groups, except for the time to operation from injury. Patients in the plate group had longer delays than the IS screw group (13.6 vs. 5.4 days, *p* = 0.001).

**Table 1:** Comparison of baseline characteristics between the plate and IS screw groups

	Plate (*n* = 14)	IS screw (*n* = 63)	*P* value
Age (years)	48.5 ± 18.1	46.0 ± 16.2	0.554
Gender			0.827
Male	8 (57.1%)	38 (60.3%)	
Female	6 (42.9%)	25 (39.7%)	
BMI (kg/m^2^)	25.2 (SD 1.9)	23.4 (SD 3.5)	0.110
Injury mechanism (number)			0.425
Traffic accident	5 (35.7%)	34 (54.0%)	
High altitude falling	5 (35.7%)	18 (28.6%)	
Crush injury	4 (28.6%)	11 (17.5%)	
Specific type of fracture (number)			**< 0.001**
Sacral dysmorphism	4 (28.6%)	0 (0%)	
Spinopelvic dissociation	9 (64.3%)	0 (0%)	
AP displacement > 10 mm	1 (7.1%)	0 (0%)	
Time to operation from injury (day)	13.5 (SD 9.8)	5.4 (SD 6.6)	**< 0.001**
Follow-up duration (month)	23.9 (SD 11.0)	39.4 (SD 34.9)	0.494

### Perioperative surgery-related data

Table 2 lists the surgery-related details. In all cases, the anterior pelvic rings were stabilized by external fixation or plating. External fixation was used more frequently than plating in both groups (78.6% in the plate group and 87.3% in the IS screw group); however, the difference was not significant (*p* = 0.410). The mean blood loss volumes were 53.0 mL in the plate group and 43.5 mL in the IS screw group, and no significant difference was observed (*p* = 0.574). The mean operation time was longer in the plate group than in the IS screw group (165.3 min vs. 95.9 min, *p* = 0.024). The length of hospital stay did not differ between the two groups (31.6 days vs. 23.2 days, *p* = 0.282).

**Table 2:** Comparison of perioperative surgery-related data and postoperative complications between the two groups

	Plate (*n* = 14)	IS screw (*n* = 63)	*P* value
Fixation method of anterior instability (n)			0.410
External fixation	11 (78.6%)	55 (87.3%)	
Anterior plating	3 (21.4%)	8 (12.7%)	
Blood loss (mL)	153.0 (SD 134.2)	43.5 (SD 21.9)	0.574
Operation time (min)	165.3 (SD 89.4)	95.9 (SD 37.7)	**0.024**
Hospital stay (day)	31.6 (SD 26.9)	23.2 (SD 18.2)	0.282
Postoperative complications (n)			
Nerve injury	0 (0%)	3 (4.8%)	> 0.999
Vessel injury	0 (0%)	0 (0%)	> 0.999
Loosening	0 (0%)	5 (7.9%)	0.578
Infection	1 (7.1%)	1 (1.6%)	0.333
Delayed union	1 (7.1%)	2 (3.2%)	0.457
Total	2 (14.3%)	11 (17.5%)	> 0.999
Revision (n)	1 (7.1%)	4 (6.3%)	> 0.999

### Postoperative complications, revision rate, and functional score

**Fig. 4:**
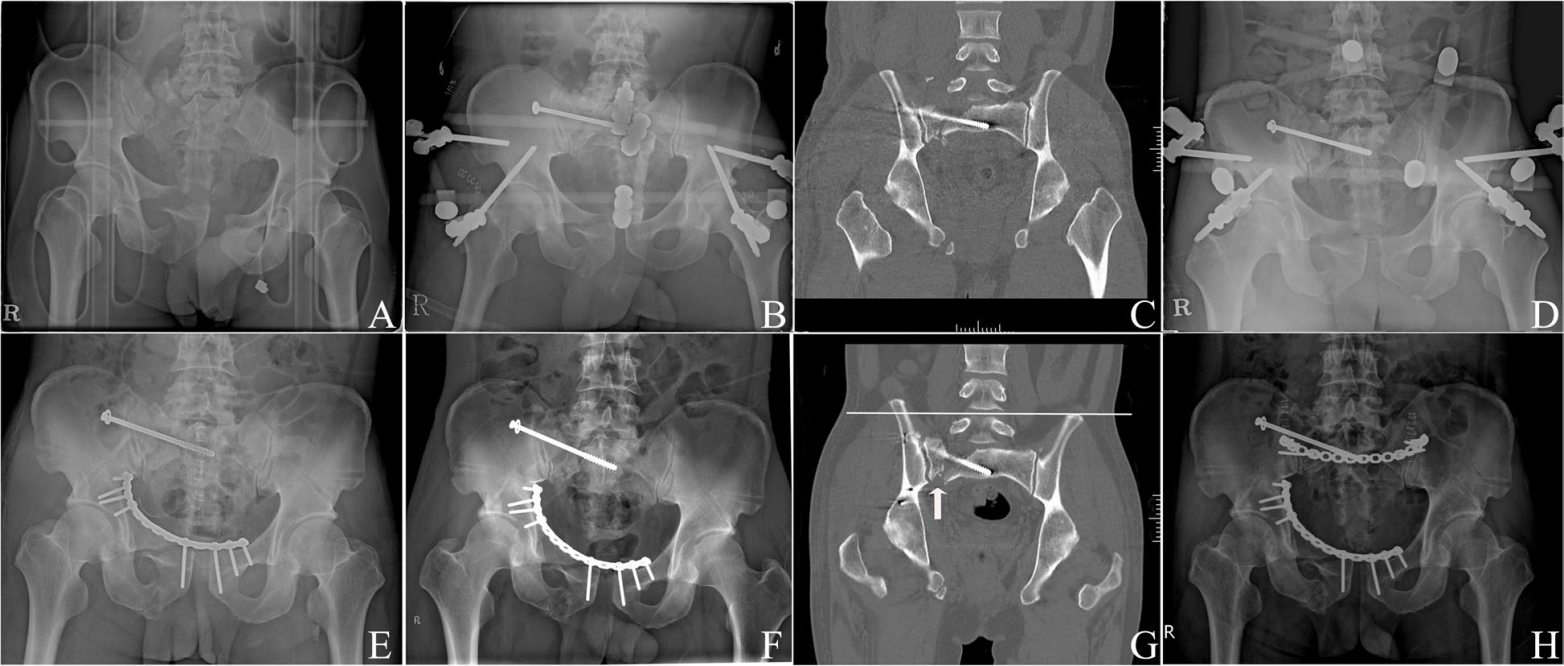
**A** A 30-year-old man with a Tile C-type pelvic ring injury. **B** External fixation and iliosacral (IS) screw fixation on the day of injury. **C** Coronal reconstructed image showing a well-reduced posterior ring. **D** Post-injury radiograph at 4 weeks showing IS screw loosening. Definitive treatment was delayed due to unstable conditions of the patient with multiple trauma including aortic dissection. **E** Anterior plating 1-month post-injury. **F** Pelvic radiograph showing IS screw migration at 8 weeks post-injury. **G** Coronal image at 8 weeks post-injury showing vertically displaced sacral alar (arrow) and upward migration of right pelvis (white line). **H** Posterior transiliac plating was performed to acquire a more rigid fixation at 2 months post-injury

In both groups, fracture union was achieved in all the patients. Three patients experienced delayed union, one from the plate fixation group (7.1%) and two from the IS screw fixation group (3.2%). In the IS screw group, 3 patients (4.8%) developed postoperative nerve injury, which was not observed in the plate group, although no significant differences were noted between the two groups (*p* > 0.999). Revision surgery for a screw change was performed in only one patient because of a screw breach to the sacral foramen, and the other two patients did not undergo screw change after confirming the absence of screw malplacement on postoperative CT. None of the groups experienced vessel injury. Five patients (7.9%) in the IS screw group experienced implant loosening, but none in the plate group (*p* = 0.578). Three of the five patients required revision surgery, two patients required additional posterior plate fixation (Fig. 4), and one required additional IS (S2) screw fixation; in two patients, no other interventions were performed as fracture union was achieved, and no additional interventions were required. One patient in each group developed surgical site infection. In the plate group, the infection was deep and was successfully treated with surgical debridement and antibiotics, while one patient with a superficial infection in the IS screw fixation group recovered after receiving intravenous antibiotics.

The overall complication rates–which included rates of delayed union, nerve injury related to posterior fixation, vessel injury, implant loosening, and surgical site infections–were 14.3% in the plate fixation group and 17.5% in the IS screw fixation group (*p* > 0.999). One patient (7.1%) in the plate fixation group and four (6.3%) in the IS screw group required revision surgery. No significant differences were found between the two groups. Additional details about perioperative surgery-related data and postoperative complications between the two groups are shown in Table 2.

The Majeed functional score only could be collected from the four patients in plate, and 34 patients in IS screw fixation group. The mean Majeed score was 76.5 points (range, 55 to 91) in plate fixation group, and 83.3 points (range, 60 to 95) in IS screw fixation group (*P* = 0.155). We could not compared the Harris hip score, due to we only could extracted the data from two in plate group (82, and 96 points), and three in IS screw group (80, 91, and 95 points).

## Discussion

Our investigation showed that the two fixation methods, percutaneous plate fixation and percutaneous IS screw fixation, were both effective treatment modalities for Tile C-type pelvic bone fractures, as supported by previous studies [3, 17, 18]. No differences were noted in terms of perioperative and postoperative complications, except for longer operation times in the plate group.

IS screw fixation was implemented in patients who required posterior fixation for Tile C-type pelvic ring injury. It is a simple and minimally invasive procedure, but it has limitations as it is difficult to perform in patients with sacral dysmorphism [19]. In addition, this approach has been associated with fixation failure in patients with vertical sacral fractures [20]. Sacral dysmorphism was found in 7–41% [21–23] of patients in previous studies, compared with 5.2% (4 patients) in our study. Although several techniques have been used to insert IS screws within a narrow safe zone, it is still technically demanding, and there is potential for screw misplacement. The navigated percutaneous screw fixation technique was introduced to redeem the surgical complications, and reducing the operation time in technically demanding C-type pelvic ring injury. Recently, the Ciolli et al. reported satisfactory results of percutaneous IS screw fixation using O-arm, combination with Stealth station navigation system [24]. However, this option is not always available, due to it needs special equipment. Triangular osteosynthesis is also one of the good options for highly unstable Tile C-type fracture recently, such as vertical shear sacral fracture [25, 26]. This technique offers mechanically better fixation compared to sole IS screw fixation, using lumbopelvic fixation from the pedicle of L5 to the ipsilateral posterior ilium. However, this technique also needs accurate IS screw fixation. In IS screw fixation, modified in-out-in corridors can enlarge safe zones, but shortening the length is inevitable, which results in weakening of the fixation power [19]. As another option, the S3 segment of a dysmorphic sacra can serve as an additional site for screw insertion [27]. Therefore, in current study we chose the plate option in these cases and achieved successful outcomes. Spinopelvic dissociation is another indication for posterior plating. Vertical displacement of the sacral alar was significantly related to IS screw failure [20], and insertion of a single IS screw would be more risky in patients with bilateral displaced sacral alar fractures. Our study demonstrated three patients with spinopelvic dissociation who achieved good clinical outcomes.

In this study, the longer operation time has been taken in plate group compared to IS screw group (*P* = 0.024). The operation time of this study includes both anterior and posterior fixation, and position change in the plate group. We believed for the posterior plate fixation, a position change (to prone position) was needed, and at least six screws were fixed, which resulted in longer operation times. In our opinion, there are also possibility of bias of our result, the surgical criteria for trans-sacral plate fixation: sacral dysmorphism, spinopelvic dissociation, and higher displacement could be more technically demanding, and this could lead to a longer surgery time compared to IS screw fixation, therefore this could be one of confounding factor for longer operation time in plate fixation group.

The current study showed no significant difference between the two groups; however, all implant loosening occurred in the IS screw fixation group. A cadaver-biomechanical study revealed that posterior trans-iliosacral plate with additional IS screw was 9% stronger at 2.5-mm displacement and 6% increased strength at 5-mm cross-headed displacement compared with a single IS screw [28]. Another biomechanical study using a pelvic bone model reported a larger displacement with the IS screw fixation technique than with the posterior tension band plating [16]. Clinical studies have also demonstrated a higher failure rate of IS screw fixation than the plate technique [12, 29, 30]. As this study showed, we recommend posterior plating in patients with vertically displaced bilateral sacral fractures or in those requiring more rigid fixation, particularly individuals with vertically displaced sacral fractures.

IS screw fixation carries a higher risk of iatrogenic nerve damage than plate fixation. Neurological complications were reported in up to 8% of patients who underwent IS screw fixation [4, 31]. The current study found no significant differences in nerve injury rates between the two methods; however, three patients experienced nerve injuries in the IS screw group. Two previous studies compared the iatrogenic nerve damage rates between the two methods. Li et al. [12] compared 13 patients who underwent plate fixation and 7 who had IS screw fixation. In this study, the rate of nerve and vessel injuries in the IS screw fixation group was twofold higher than that in the plate fixation group. Chen et al. [2] reported 2 patients who had nerve injury among the 29 patients from the IS screw fixation group, while none experienced such injury in the percutaneous posterior plate fixation group with a sample size of 29 patients. However, neither of the studies found any significant differences between the two groups, perhaps due to the small sample size. Our study had a larger sample size; however, the results seemed comparable to those of previous studies. In this study, the incidence of iatrogenic nerve injuries was 4.8%. More caution should be applied, and careful examination of fluoroscopic images must be performed [32]. The use of navigation techniques for guidewire placement and intraoperative 3D-image control of the guidewire position are helpful in reducing this complication [33, 34].

There is a consensus that longer operation times are disadvantageous because they lead to higher rates of postoperative infection [35]. This is relevant to our findings regarding operation times in the two treatment groups. In current series, we found longer operation time in plate group, Moreover the rate of wound infection was 7.1% in the plate group, while it was only 1.6% in the IS screw group. Fortunately, the patients in the plate group who developed infections only required surgical debridement. Posterior plate fixation requires posterior soft tissue dissection in thin layers, which can put a patient at risk of developing infection. We attempted to place the plate below the PSIS level to minimize the irritation caused by the plate, and a small skin incision was made to carefully dissect the soft tissue and perform tunneling.

A number of studies have investigated the surgical outcomes of posterior plating using a 4.5-mm plate; however, a 3.5-mm reconstruction plate could minimize soft tissue irritation. Previous studies have discussed the occurrence of wound infections following posterior plate fixation, with reported rates of 10.5–30% [3, 36, 37]. The study analyzed the group that underwent insertion of a 4.5-mm plate above the PSIS, which required a longer incision compared with the other group and had a 17.2% (5 of 29) infection rate with all patients who developed infections requiring surgical debridement [36, 37]; meanwhile, one study that involved the insertion of a 3.5-mm plate below the PSIS showed a 4.8% deep infection rate [3]. A 3.5-mm reconstructed plate was placed below the PSIS and required a small incision, which showed satisfactory results without mechanical failures. In the current study, although we tried to prevent the occurrence of wound complications, one patient with multiple severe traumatic injuries developed an infection due to prolonged bed rest. However, only one patient had postoperative infection (7.1%), which is a relatively lower rate compared with that reported in previous studies that used a 4.5-mm plate for posterior plate fixation. Therefore, we recommend a 3.5-mm reconstruction plate placed below the PSIS to minimize irritation and wound complications. Surgeons should pay close attention to the risk of surgical wounds in patients undergoing posterior transiliac plating.

This study has several limitations. First, the sample size was relatively small; however, to the best of our knowledge, this study has the largest number of study participants to date in order to compare posterior transiliac plate fixation and IS screw fixation. Second, there was an imbalance in the number of patients in the two groups; the plate fixation group had a smaller number of patients than the IS screw group. It is because the indication of plate fixation were sacral dysmorphism, spinopelvic dissociation with bilateral sacral alar fractures, or a severe AP displacement over 10 mm. In real-world, these injuries is not a common situation, therefore we believe the current study could be a meaningful result even the number of patients on each groups were different. Third, we could not fully compare the patient-reported outcome measures (PROM), only part of data could be extracted because of the retrospective nature of the study, and its study population mainly consisted of severely injured patients. Further studies with larger sample sizes and more detailed outcome variables, such as PROM, or well-structured synthetic analysis are needed to draw more definitive conclusions on this topic.

## Conclusions

We recommend IS screw fixation as the primary treatment for posterior pelvic ring fixation in patients with Tile C-type pelvic fractures. Surgeons should consider the risk of screw loosening and iatrogenic nerve injuries. Based on the results of this study, we recommend percutaneous posterior transiliac plate fixation for patients with 1) sacral dysmorphism and 2) bilateral sacral alar fractures (spino-pelvic dissociation) that require a more rigid fixation, including revision.

## Data Availability

The datasets generated during and analyzed during the current study are not publicly available due to it contains potentially identifying information of each patient but are available from the corresponding author on reasonable request.

## Abbreviations

IS: Iliosarcral
AP: Anteriorposterior
PSIS: Posterior superior iliac spine
SD: Standard deviation
PROM: Patient-reported outcome measures
